# Medical News and Miscellany

**Published:** 1887-06

**Authors:** 


### MEDICAL NEWS AND MISCELLANY.

"THE OLD RELIABLE."—The Medical Department of the Tulane University, formerly University of Louisiana,—"the old school,"—held its *fifty-third* annual commencement March 30, 1887. Eloquent addresses were delivered by Bishop Gallagher and by the valedictorian, Dr. A. A. Forsythe, also one by Prof. Chaille, in memory of the philanthropist, Paul Tulane. Hon. Jefferson Davis was present. We observe in the catalogue sent us by the Dean, Prof. S. E. Chaille, that the following Texans graduated M. D., on that occasion. J. J. Atkinson, A. T. B. Beauchamp, A. Beckman, J. M. Burford, A. D. Duncan, C. L. Geyer, J. W. Hamilton, F. M. D. Hill, J. F. Johnson, D. F. Kirkpatrick, L. F. Layton, J. A. Mercer, A. L. Montgomery, Irwin Pope, J. W. Scott, J. R. Smith, 16.

The system of instruction in this famous school is thorough, and the clinical advantages offered by the great Charity Hospital are unsurpassed by any in America. A diploma from this College is justly, very highly prized and carries with it, respectability of a high order.

---

ANOTHER PRESS VICTIMIZER.—The Cherry Malt concern, the "Liebig Parmacal Company," of New York, made a written contract with us for $20 advertising. On presentation of first bill, payment was refused, and they persistently refuse to answer letters on the subject. They have beat us out of five months advertising of Cherry Malt and we throw this in for the benefit of the concern, to make it even money, and charge the whole to *profit and loss.* Look out for them.

---

DR. STONE NOT DEAD.—Our information that Dr. T. M. Stone, of Jasper, had died September 14, 1886, of Pneumonia, as announced in THE JOURNAL for April proves to be incorrect. Mrs. Stone died on that date. We presume our informant was misled by that fact. We are much gratified to be able to make the announcement.

---

INTERNATIONAL CONGRESS ON INEBRIETY.—The Council of the English Society for the study and cure of inebriety, have completed arrangements for an International Medical Congress, to be held at Westminster Hall, London, *July 5th and 6th, 1887.*

The object of this Congress is to present and discuss the problems of inebriety medically, and from a purely *scientific standpoint,* by the best authorities, thus laying the foundation for a broader and more exact study of this subject.

---

YELLOW FEVER THREATENING TEXAS.—Yellow Fever has made its appearance at Key West, Fla., and a number of deaths have occurred. New Orleans promptly quarantined, and Texas as promptly followed suit. The former requires five days detention which did not suit State Health Officer Rutherford who demanded ten, which being refused, New Orleans was included in the embargo; and now all persons coming from New Orleans are "inspected."

The occurrence of Yellow Fever on the Southern Coast thus early, is serious cause of apprehension, and we commend the prompt action of the Health Officer in quarantining against the infected places; but, unless the fever were *epidemic* in New Orleans and the atmosphere infected, a rigid quarantine against that city would seem to be unnecessary. We are sorry to see State Health Officer Rutherford and President Holt lock horns so early — but we must back our own man, every time.

A NEW DISTRICT SOCIETY TO BE ORGANIZED.—A number of physicians in Austin and vicinity are agitating a proposition to organize a District Medical Society, at an early date; and a call will be issued for a meeting, before many days. Several plans are proposed, but the idea that seems to meet most favor is, to build the new association on the Travis County Medical Society, which will then be incorporated; or in other words, to develop the present organization, the Travis County Medical Society into a District Society. We invite the attention of all physicians residing in counties contiguous to Travis county to the subject and ask their co-operation, in the laudable enterprise. A circular letter will be sent out inviting a meeting.

